# LINC00669 insulates the JAK/STAT suppressor SOCS1 to promote nasopharyngeal cancer cell proliferation and invasion

**DOI:** 10.1186/s13046-020-01674-z

**Published:** 2020-08-24

**Authors:** Xiang Qing, Guo-lin Tan, Huo-wang Liu, Wei Li, Jin-gang Ai, Shan-shan Xiong, Meng-qing Yang, Tian-sheng Wang

**Affiliations:** 1 Department of Otolaryngology Head and Neck Surgery, The Third Xiangya Hospital, Central South University, Changsha, 410013 China; 2 Department of Postgraduate Office, Third Xiangya Hospital, Central South University, Changsha, 410013 China

**Keywords:** Nasopharyngeal carcinoma, Long non-coding RNA, LINC00669, JAK/STAT, SOCS1, STAT1

## Abstract

Nasopharyngeal carcinoma (NPC) is an epithelial cancer emerging from the lining of nasopharyngeal mucosa, with extremely frequent occurrence in east and southeast Asia. For the purpose of exploring roles of the dysregulated long non-coding RNA (lncRNA) in NPC, we identified a novel lncRNA LINC00669 with an apparent negative correlation to the overall survival from human NPC mRNA expression profiling databases. We further performed RNA pulldown coupled with mass spectrum to find out its target protein, and applied a series of in vitro and in vivo loss-and-gain-of function assays to investigate its oncogenic roles in NPC tumor development and progression. Our results demonstrated that LINC00669 competitively binds to the key JAK/STAT signaling pathway suppressor SOCS1, and insulates it from imposing ubiquitination modification on the pathway component of STAT1, which leads to its abnormal stabilization and activation. The activated STAT1 is then transferred into the nucleus and initiates the transcription of genes related to proliferation and invasion. In summary, our study reveals that the cytoplasmic resident lncRNA LINC00669 confers malignant properties on NPC cancer cells by facilitating a persistent activation of the JAK/STAT signaling pathway. Findings in the current study shed lights on prospects for treating NPC using strategies targeting the novel regulator of the JAK/STAT signaling.

## Introduction

Nasopharyngeal carcinoma (NPC) is one of the most frequent malignant tumors with extremely high occurrence in Southern China, Northern Africa and parts of the Mediterranean basin [1]. Most NPC is moderately sensitive to radiation therapy, which has been the preferred treatment thus far [2]. However, NPC is featured as a type of poor or undifferentiated carcinoma with only 41–63% overall survival rate in patients at the advanced stage [3, 4]. Therefore, deeper insights into the molecular mechanisms that underlie NPC development and progression warrant the better therapeutic strategies targeting the disease.

Long non-coding RNA (lncRNA) is a class of longer than 200 nucleotides RNA transcripts [5]. Emerging studies have shown that lncRNAs participate in NPC development and progression. For example, maternally expressed gene 3 (MEG3) was identified as a tumor suppressor lncRNA that is downregulated in NPC due to losses of DNA copy numbers and aberrant promoter methylation [6]. On the contrary, LncRNA HOX transcript antisense intergenic RNA (HOTAIR) acts as an oncogene, whose high abundance in NPC cells and the patient tumor samples correlates with a poor prognosis [7, 8]. With the advance of microarray and high-throughput RNA sequencing, a large number of lncRNAs have been identified in NPC tissues and cell lines [9]. However, the roles of these dysregulated lncRNAs in the pathogenesis of NPC remain poorly understood.

The Janus kinase (JAK)-signal transducer of activators of transcription (STAT) signaling pathway is involved in diverse biological processes such as cell proliferation, differentiation, apoptosis, and immune regulation [10]. It mainly consists of three components, namely tyrosine-kinase-related receptor, tyrosine kinase JAK and transcription factor STAT. The binding of cytokines to their receptors activates JAKs and promote STAT nuclear translocation to initiate the corresponding gene transcription, including the expression of SOCS which feeds back negatively to shut down the signaling pathway [11]. It was reported that the abnormal JAK/STAT signaling with persistent activation of STAT1, 3 and 5 has a cancer-promoting role in the progression of Epstein-Barr virus (EBV)-related NPC [12]. Therefore, suppression of the aberrantly activated JAK/STAT signaling pathway could be promising strategy for NPC therapy.

In current study, we demonstrated an abnormal accumulation of lncRNA LINC00669 in the cytoplasm of NPC cells, where it binds to the key JAK/STAT signaling pathway suppressor SOCS1 and blocks its ubiquitin ligase activity toward the transcription factor STAT1. The stabilized STAT1 is further activated and translocated into the nucleus to initiate expression of genes associated with proliferation and invasion. These results shed light on a clinical implication of LINC00669 as a diagnostic and prognostic marker as well as a therapeutic target of NPC.

## Materials and methods

### Ethical statement

All animal experiments were conducted in accordance with the guidelines of Animal Experimentation Ethics Committee of the Third Xiangya Hospital of Central South University. Clinical NPC tumor and para-tumor samples were collected from 16 patients with informed consents being signed by all of the patients.

### Cell lines and treatment

All the NPC cell lines and normal human nasopharyngeal epithelial cell line NP69 were purchased from the Chinese Academy of Sciences Cell Bank (Shanghai, China). NPC cells were cultured in Dulbecco’s modified Eagle’s medium (Gibco, Thermo Fisher Scientific, Inc., Waltham, MA, USA) containing 10% fetal bovine serum (FBS) (Sigma-Aldrich, St. Louis, MO, USA) and 1% antibiotics (100 U/ml penicillin and 100 mg/ml streptomycin sulfates), while NP69 cells were maintained in the RPMI1640 medium containing 10% FBS and 1% penicillin/streptomycin. Cycloheximide (20 mg/ml, Millipore Sigma, #C7698) and MG132 (5 μM, Sigma-Aldrich, #1211877-36-9) were used for studying ubiquitin-proteasome-mediated protein degradation.

### Nucleic acid extraction and mRNA expression quantification

Total RNA of NPC tissues and cells was extracted using TRIzol reagent (Invitrogen; Thermo Fisher Scientific, Inc.), followed by reverse transcription using RevertAidTM H Minus First Strand cDNA Synthesis Kit (Fermentas). The relative mRNA contents were quantified by quantitative PCR (qPCR) using SYBR Green PCR Master Mix (ABI 4309155) in the ABI7900 Realtime-PCR machine. Primers used for qPCR analysis were included in Table 1.

**Table 1:** Primer list

Primers for qPCR	
Genes	Primer sequences (5′--3′)
Primers U1 -F	GGGAGATACCATGATCACGAAGGT
Primers U1 -R	CCACAAATTATGCAGTCGAGTTTCCC
JAK2-F	ATCCACCCAACCATGTCTTCC
JAK2-R	ATTCCATGCCGATAGGCTCTG
JAK3-F	CCTGATCGTGGTCCAGAGAG
JAK3-R	GCAGGGATCTTGTGAAATGTCAT
STAT1-F	CAGCTTGACTCAAAATTCCTGGA
STAT1-R	TGAAGATTACGCTTGCTTTTCCT
STAT3-F	ATCACGCCTTCTACAGACTGC
STAT3-R	CATCCTGGAGATTCTCTACCACT
SOCS1-F	TTTTCGCCCTTAGCGTGAAGA
SOCS1-R	GAGGCAGTCGAAGCTCTCG
LINC00669-F	CAGTGAGATGCAGAGCTTGG
LINC00669-R	TGTCCTTGAGGCTGTCTGTG
β-actin -F	CATGTACGTTGCTATCCAGGC
β-actin -R	CTCCTTAATGTCACGCACGAT
**Oligonucleotide sequence for RNA pulldown**	
LINC00669-sense-F	**TAATACGACTCACTATAGGGAGA**CTGTGTGGAACTTGGGATTCGAACGG
LINC00669-sense-R	CAATTTTGATTGGCTTTATTTTGATGTG
LINC00669-antisense-F	CTGTGTGGAACTTGGGATTCGAACGG
LINC00669-antisense-R	**TAATACGACTCACTATAGGGAGA**CAATTTTGATTGGCTTTATTTTGATGTG

### Gene cloning, knockdown and overexpression

The truncated LINC00669 plasmids were constructed using Phusion Site-Directed Mutagenesis Kit (Thermo Fisher Scientific, Inc., Waltham, MA, USA) by following the manual instruction. In vitro loss-of-function studies were performed by pre-designed siRNA from Millipore Sigma using Lipofectamine™ 2000 (Invitrogen; Thermo Fisher Scientific, Inc.). For in vivo xenograft experiment, lentiviral vectors expressing shLINC00669, SOCS1, shSOCS1 and the empty lentiviral vector were purchased from GeneChem (Shanghai, China for the stable infection of CNE-2 cells upon puromycin (2 μg/ml) selection.

### Western blotting

Total proteins from NPC tissues and cells were extracted using RIPA buffer (1% NP-40, 0.1% SDS, 50 mM DTT) supplemented with protease inhibitor cocktail containing 2 μg/ml Aprotinin, 2 μg/ml Leupeptin and 1 mM PMSF. An equal amount of total protein from each sample was separated by 8 ~ 15% SDS-PAGE gel and electroblotted onto a polyvinylidene difluoride membrane. Immunoblotting was conducted using antibodies listed in Table 2.

**Table 2:** Antibody list

Primary antibodies	MW (kDa)	Dilution	Company / Catalog	Secondary antibodies	Dilution
SOCS1	≈38	1:500	Santa Cruz, sc-518,028	Goat Anti Rabbit IgG/HRP	1:4000
JAK2	≈120	1:500	omnimabs,OM206683	Goat Anti Rabbit IgG/HRP	1:4000
JAK3	≈125	1:1000	omnimabs,OM260121	Goat Anti Mouse IgG/HRP	1:4000
STAT1	≈87	1:1000	Abcam,ab234400	Goat Anti Rabbit IgG/HRP	1:4000
STAT3	≈88	1:2000	Abcam,ab119352	Goat Anti Mouse IgG/HRP	1:4000
p-STAT1(Y701)	≈87	1:1000	Abcam,ab29045	Goat Anti Mouse IgG/HRP	1:4000
UB	≈8.4	1:200	Ptgcn,10,201–2-AP	Goat Anti Rabbit IgG/HRP	1:4000
HIS		1:500	Abcam,ab18184	Goat Anti Mouse IgG/HRP	1:4000
GFP		1:1000	Abcam,ab290	Goat Anti Rabbit IgG/HRP	1:4000
FLAG		1:1000	Abcam,ab205606	Goat Anti Rabbit IgG/HRP	1:4000
β-actin	42	1:2000	Ptgcn, 66,009–1-Ig	Goat Anti Mouse IgG/HRP	1:4000

### Co-immunoprecipitation (Co-IP)

Total proteins from NPC cells cultured in 10-cm culture dishes were used for Co-IP assay. Briefly, primary antibodies against SOCS1 or STAT1 were added in each cell lysate and incubated overnight at 4 °C with rotation. The normal human IgG protein was used as a negative control. Protein A/G agarose beads (Beyotime Biotechnology, #P1012) were then added for affinity binding of primary antibody by a 2 h-incubation at 4 °C with gentle rotation. The unbound antibodies were washed off through the sequential wash with PBS and cell lysis buffer. The agarose beads were resuspended in 20 μl 1x SDS-PAGE loading buffer, and boiled for western blot analysis of the precipitated target proteins.

### Cell proliferation assay

Time-dependent NPC cell proliferation was characterized by MTT cell proliferation and cytotoxicity detection kit (KeyGEN Biotech, #KGA312) by following the manual instruction.

### Wound-healing assay

NPC cell migration was characterized by wound-healing assay. Briefly, a confluent monolayer of NPC cells in 24-well plate was scratched using a sterile 100 μl pipette tip. Cells were then allowed to expand in FBS-reduced growth medium (1%) for another 48 h. The migration was assessed using the scratch ratio by dividing the interval at the starting point (0 h) with the one at the ending point (48 h).

### Transwell assay

NPC cells were seeded into the Matrigel (BD Biosciences, San Jose, CA)-coated upper chamber of 8.0 μm pore size Transwell apparatus (Corning, NY, USA) with serum-free medium. Growth medium supplemented with 10% FBS was added to the lower chamber as a chemoattractant. The cells were allowed to invade for 48 h at 37 °C under 5% CO_2_. Cells invaded to the lower surface of filter were then fixed in 70% ethanol for 30 min followed with staining by 0.1% crystal violet for 10 min at room temperature.

### Fluorescence in situ hybridization (FISH)

Cellular LINC00669 localization was characterized using the fluorescence in situ hybridization kit from Ribo Bio (Guangzhou, China) according to the manufacturer’s instruction.

### Cell apoptosis

NPC cell apoptosis were determined by flow cytometry using Annexin V-PE/7-AAD Apoptosis Kit (Abnova, #KA3809) according to the manual instruction.

### Immunohistochemistry (IHC)

Tissues for IHC were fixed in 10% buffered formalin for 24 h and embedded in paraffin. The deparaffinized and rehydrated sections were blocked for endogenous peroxidase by incubation in 3% hydrogen peroxide followed with antigen retrieval in the boiling citrate buffer (10 mM, pH 6.0) for 10 min. The sections were then blocked with normal goat serum (1:10) and subject to incubation with anti-Ki67 monoclonal antibody (1:100, Dako, Glostrup, Denmark) or anti-Caspase 3 antibody (1:100, Abcam, #ab32351) overnight at 4 °C. Thereafter, the PBS cleaned sides were incubated with the biotinylated secondary antibody at 37 °C for 30 min, and subsequently incubated with a 1:200 streptavidin-biotin-peroxidase complex (Sigma, St. Louis). Reactive products were visualized with 3,3′-diaminobenzidene (DAB) as the chromogen, and nuclei were counter-stained with hematoxylin.

### Immunofluorescence

NPC cells were fixed with 4% paraformaldehyde and permeabilized by 0.2% Triton X-100. After 1 h blocking with 1% bovine serum albumin at room temperature, the cells were incubated with anti-STAT1 (1:100) antibody overnight at 4 °C. Next day, the cells were washed off the unbound primary antibody and stained with donkey anti-rabbit IgG Alexa Fluor 488 secondary antibody (ThermoFisher, #A32790) for 1 h at room temperature.

### In vivo xenograft experiments

Male BALB/c nude mice (8-week-old, *n* = 6 mice/group) from Beijing HFK Bioscience Co. Ltd. (Beijing, China) were maintained under pathogen-free conditions. After subcutaneously injected with 10^6^ control or viral shRNA infected CNE-2 cells, the mice were monitored for tumor growth from 1 week after injection. Tumor size was calculated based on the formula of Volume = a x b^2^ × 0.52 (a, long diameter; b, short diameter).

### RNA pulldown assay

Biotin-labeled full-length LINC00669 and antisense LINC00669 were synthesized in vitro using Biotin RNA Labeling Mix (Roche) and the Riboprobe Systems with T7 RNA polymerase (Promega). After DNase I treatment, the RNA probes were purified with RNeasy Mini Kit (QIAGEN). For each pulldown assay, 30 μg of biotin-labeled RNA probes were incubated with CNE-2 cell lysates at room temperature for 4 h followed by adding pulling down the binding protein partner with streptavidin magnetic beads (TermoFisher, USA) at 4 °C overnight. The proteins were then separated by electrophoresis and visualized with sliver staining. The unique bands pulled down by sense LINC00669 were subject to mass spectrometry and retrieved in human proteomic library.

### RNA immunoprecipitation (RIP)

RIP assay was conducted in wildtype and LINC00669-depleted NPC cells using Magna RIP RNA-binding protein immunoprecipitation Kit (Millipore, MA) according to manufacturer’s instruction. Antibodies against the normal human IgG and SNRNP70 were used as negative and positive controls for RIP, respectively. The relative enrichment of LINC00669 was normalized to the amount of the enriched U1snRNA.

### Bioinformatics

The common signatures in NPC were searched by overlapping the upregulated genes (*p* ≤ 0.05, log2 fold change ≥2) in databases of GSE12452 and GSE53819 followed by the overall survival correlation analysis using the Signature-based statistics tool in GEPIA 2 (http://gepia2.cancer-pku.cn/#index). In silico prediction of LINC00669-SOCS1 interaction was performed using online tools from catRAPID (http://s.tartaglialab.com/page/catrapid_group).

### Statistical analysis

Data are expressed as the mean ± standard deviation of three independent experiments. Two-tailed Student’s *t*-test was performed for detecting difference between two groups, while One-Way ANOV test was conducted for comparing two-group difference among the multiple groups using GraphPad Prism 6 software. *P* < 0.05 was considered as significant.

## Results

### LINC00669 is upregulated in NPC tumors and cells

**Fig. 1:**
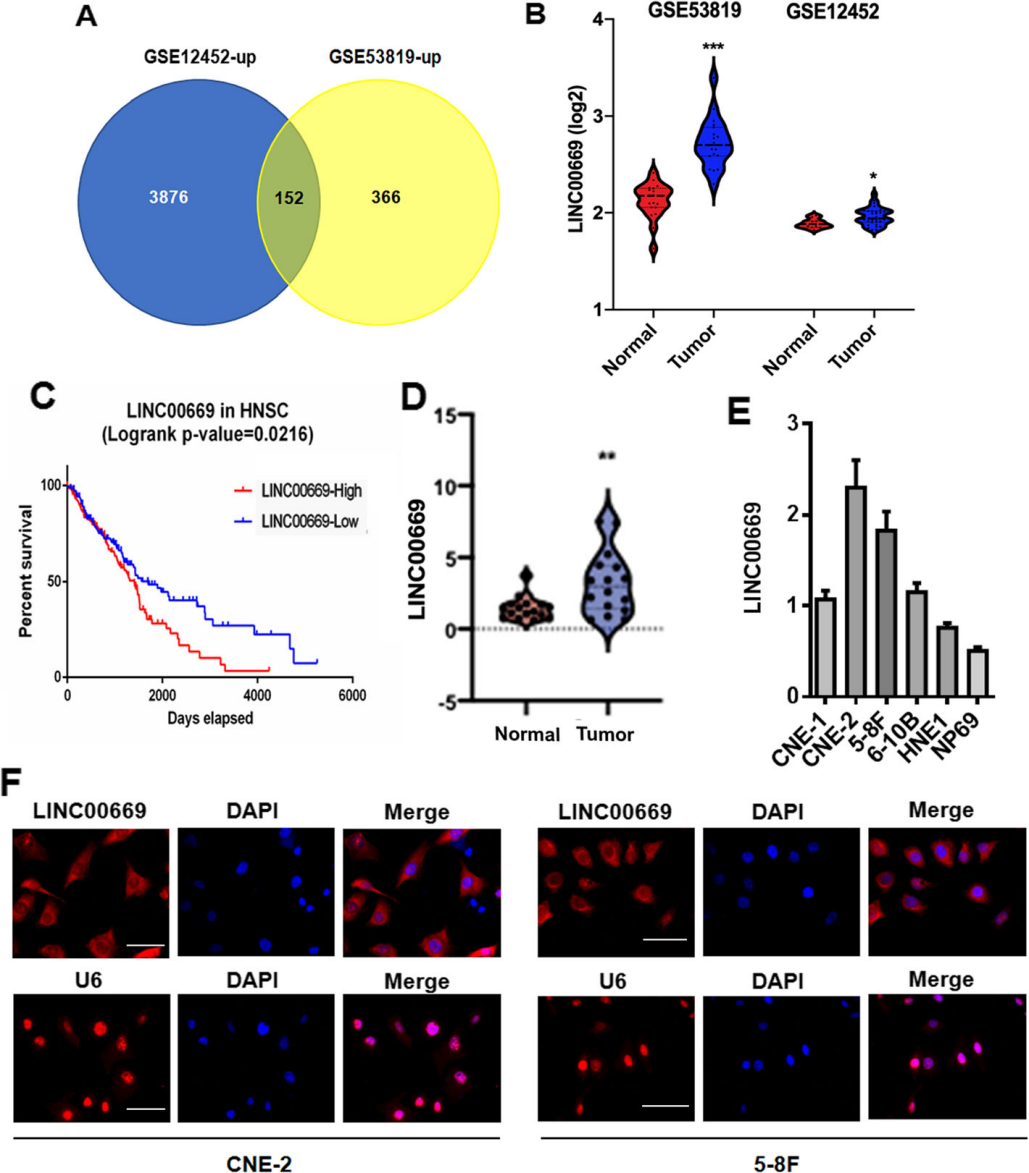
Aberrant expression of LINC00669 in NPC tumors and cells. **a**, Venn diagram showing 152 common lncRNA signatures that are upregulated in NPC mRNA expression profiling databases of GSE12452 [14] and GSE53819 [15]. **b**, Microarray data showing upregulation of LINC00669 in NPC tumors. **c**, Kaplan-Meier analysis showing a negative correlation of the overall survival rate of NPC patients with LINC00669 level. **d**, qPCR analysis showing an upregulation of LINC00669 in the clinical NPC tumor samples. ** *p* < 0.01. **e**, qPCR analysis showing a general upregulation of LINC00669 in different NPC cell lines. **f**, FISH analysis showing cytoplasmic localization of LINC00669 in NPC cells. DAPI was used for nuclear counterstaining. U6 was used as a positive control for nuclear localization

Emerging roles of lncRNA in cancer development and therapeutic opportunities have aroused wide research interest and attention [13]. To explore novel lncRNAs that play key roles in NPC, we retrieved and interrogated the expression profiles of lncRNAs in two independent NPC mRNA expression profiling databases of GSE12452 [14] and GSE53819 [15] to have identified 153 commonly upregulated signatures (Fig. 1a). None of the other lncRNAs showed significant difference in the survival curve analysis except for three classical lncRNAs of LINC00669, AFAP1-AS1 and CT75, whose upregulation are negatively correlated to the overall survival of NPC patients (Fig. 1b, c). Since LINC0069 was a novel lncRNA whose function was completely unknown, we made it the focus in our current study. Consistently, LINC00669 was confirmed by qPCR to be significantly upregulated in the patient-derived NPC tumors (Fig. 1d) and a panel of the general NPC cell lines (Fig. 1e) in comparison to their corresponding controls. Due to the highest expression of LINC00669 in CNE-2 and 5-8F among the tested cell lines, they were chosen for the subsequent functional studies. FISH assay showed that LINC00669 is dominant in the cytoplasm in both CNE-2 and 5-8F cells (Fig. 1f), indicating the potential roles involved in translational or signaling regulations.

### LINC00669 confers malignant properties to NPC cells

**Fig. 2:**
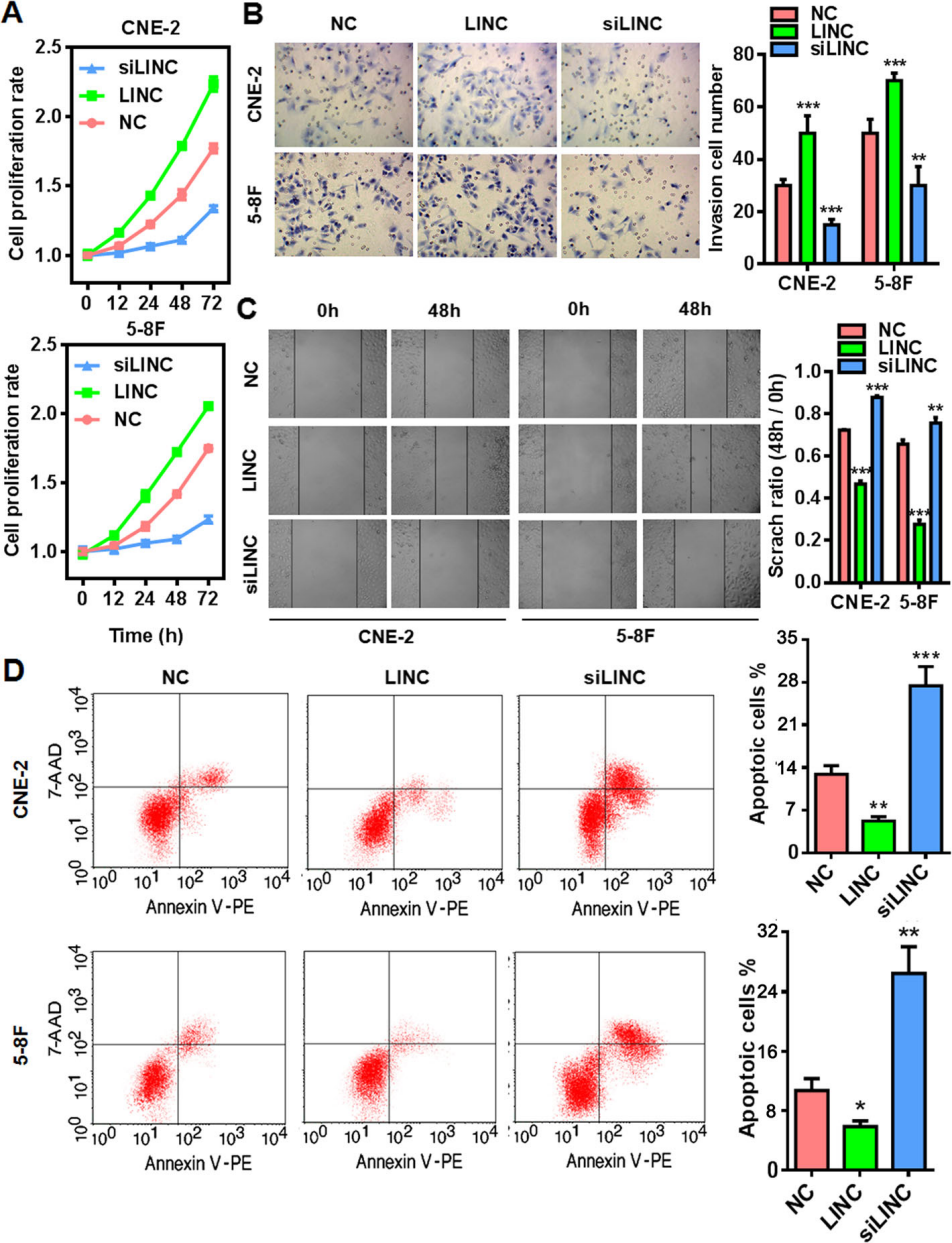
LINC00669 plays oncogenic roles in NPC cells. **a**, MTT assay showing the stimulative effect of LINC00669 on the time-dependent proliferation in NPC cells. NC, negative control; siLINC, siRNA-mediated silencing of LINC00669; LINC, ectopic expression of LINC00669. **b**, Transwell assay showing the invasion-promoting effect of LINC00669 in NPC cells. ** *p* < 0.01, *** *p* < 0.001 versus NC group. **c**, Wound-healing assay showing the migration-promoting effect of LINC00669 in NPC cells. ** *p* < 0.01, *** *p* < 0.001 versus NC group. **d**, Representative flow cytometry plots showing the pro-survival effect of LINC00669 in NPC cells. Apoptotic cells were featured by Annexin V positive but 7AAD negative staining pattern. * *p* < 0.05, ** *p* < 0.01, *** *p* < 0.001 versus NC group

Next, we conducted gain- and loss- of function assays to explore the functions of LINC00669 in NPC. Consistent in both CNE-2 and 5-8F cell lines, forced expression of LINC00669 dramatically promoted cell proliferation (Fig. 2a), invasion (Fig. 2b) and migration (Fig. 2c), while its depletion by small interfering RNA (siRNA) exhibited the opposite effects. Meanwhile, LINC00669 is essential for the survival of NPC cells as indicated by the strikingly induced spontaneous apoptosis upon loss of LINC00669 (Fig. 2d). Together, these data indicated that LINC00669 profoundly endows carcinogenic properties of NPC cells.

### LINC00669 physically interacts with SOCS1

**Fig. 3:**
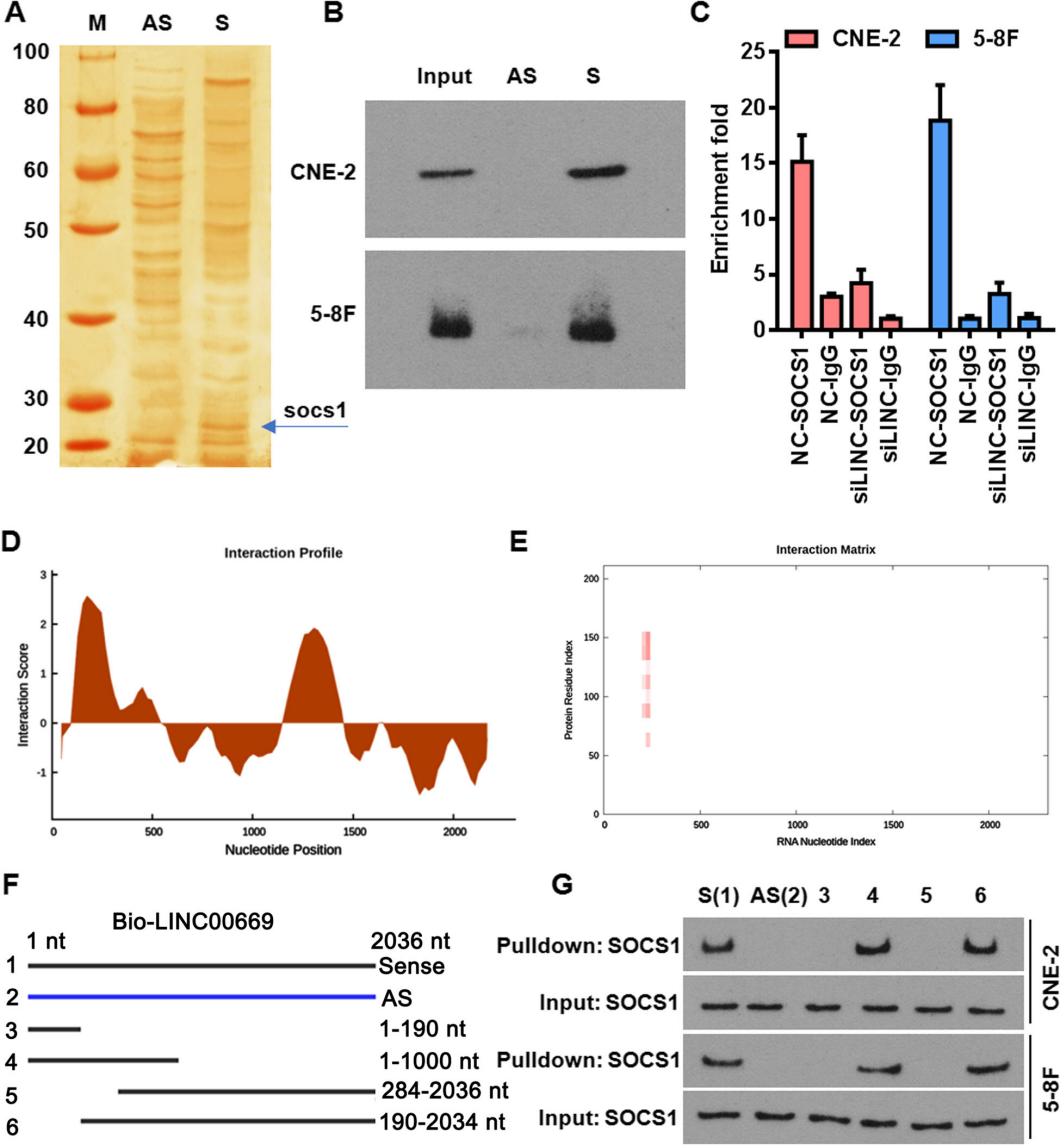
LINC00669 physically binds to SOCS1. **a**, Silver staining of the proteins pulled down from LINC00669 sense and antisense RNAs incubated CNE-2 cell lysates. AS, antisense; S, sense. **b**, Western blotting confirming the interaction between LINC00669 and SOCS1 in NPC cells. **c**, qPCR analysis of LINC00669 enrichment in SOCS1-immunoprecipitated RNAs in control and LINC00669-depleted NPC cell lysate. Antibody against normal human IgG was included as a RIP negative control. **d** and **e**, interaction profile (**d**) and interaction matrix (**e**) demonstrating catRAPID fragments-based prediction of interaction between LINC00669 and SOCS1. **f**, Schematic diagram showing construction of the truncated LINC00669. **g**, Western blotting showing only the truncated LINC00669 constructs encompassing 190–283 nt domain were able to pulldown SOCS1

Inspired by the cytoplasmic localization of LINC00669 (Fig. 1e), we speculated that it might participate in the translational or signaling regulations. To identify its target proteins, we incubated CNE-2 cell lysates with a biotin-labeled LINC00669, and proceeded with electrophoresis followed by silver staining. Mass spectrum analysis of the unique protein bands detected only in the sense but not anti-sense LINC00669-incubated cell lysates by silver staining indicated SOCS1 as a potential LINC00669-targeting protein (Fig. 3a). The direct interaction between LINC00669 and SOCS1 was further confirmed by western blot on LINC00669 pulldown cell lysates using an antibody against SOCS1 (Fig. 3b). Vice versa, RNA-immunoprecipitation (RIP) assay using SOCS1 antibody followed by qPCR analysis demonstrated that SOCS1 specifically precipitated LINC00669 in both CNE-2 and 5-8F cell lysates, and the enrichment of LINC00669 was largely decreased upon siRNA-mediated depletion of LINC00669 (Fig. 3c).

Next, we conducted an *in-silico* prediction through catRAPID [16] to map the domain in LINC00669 that mediates its interaction with SOCS1. The analytical results of both interaction profile (Fig. 3d) and interaction matrix (Fig. 3e) suggested that RNA region of 190–283 nucleotides (nt) and the protein domain ranging from 76 to 187amino acids in SOCS1 were mostly likely to mediate the mutual binding (Table 3). Accordingly, we made a series of the truncated LINC00669 constructs flanking the site of 190–283 nt for testing their binding potentials with SOCS1 (Fig. 3f). Consistent with the predication by catRAPID, only the constructs encompassing region of 190–283 nt were able to pulldown SOCS1 (Fig. 3g). Taken together, these data indicated SOCS1 is an immediate target of LINC00669 for executing its oncogenic functions in NPC cells.

**Table 3:** Domains mediating the mutual interaction between LINC00669 and SOCS1 predicted by catRAPID

#	Protein region	RNA region	Interaction Propensity	Discriminative Power	Normalized Score
**1**	**136–187**	**190–283**	**11.47**	**33**	**2.82**
**2**	**126–177**	**190–283**	**11.36**	**33**	**2.80**
**3**	**76–127**	**190–283**	**10.54**	**32**	**2.69**
**4**	**101–152**	**190–283**	**10.18**	**32**	**2.64**
**5**	126–177	185–278	9.80	28	2.59
**6**	51–102	190–283	9.39	28	2.53
**7**	136–187	185–278	9.38	28	2.53
**8**	76–127	185–278	9.32	28	2.52
**9**	101–152	185–278	8.48	26	2.40
**10**	86–137	190–283	8.22	26	2.37
**11**	111–162	190–283	8.06	26	2.35
**12**	51–102	185–278	7.97	24	2.33
**13**	136–187	231–324	7.86	24	2.32
**14**	36–87	190–283	7.59	24	2.28
**15**	101–152	231–324	7.23	24	2.23
**16**	111–162	185–278	7.15	24	2.22
**17**	36–87	185–278	7.12	24	2.22
**18**	26–77	190–283	7.03	24	2.20
**19**	86–137	185–278	7.02	24	2.20
**20**	26–77	185–278	6.17	22	2.08

### SOCS1 influences various characters of NPC cells by negatively regulates the JAK/STAT signaling pathway

**Fig. 4:**
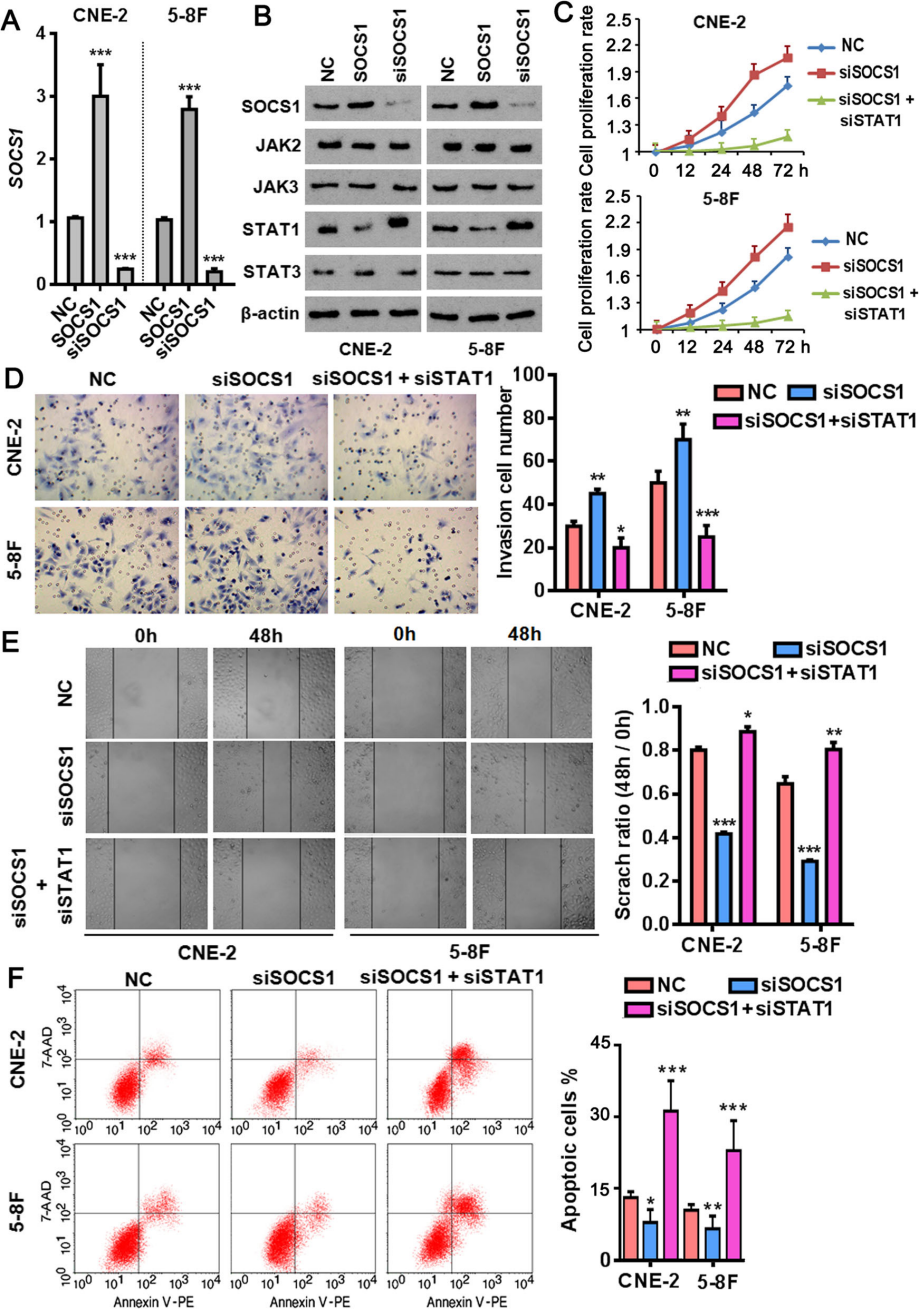
SOCS1 plays as a tumor suppressor in NPC cells by regulating STAT1 protein. **a**, qPCR analysis confirming ectopic expression (SOCS1) and knockdown of SOCS1 (siSOCS1) in NPC cells. *** *p* < 0.001 versus negative control (NC) group. **b**, Western blotting showing the effects of SOCS1 in the expression of the JAK/STAT signaling pathway component proteins. STAT1 was decreased when overexpressing SOCS1, while was increased when silencing SOCS1. **c**, MTT assay showing the inhibitory effect of SOCS1 on the time-dependent proliferation in NPC cells, which could be partially rescued upon the simultaneous depletion of STAT1. NC, negative control; siSOCS1, siRNA-mediated silencing of SOCS1; siSTAT1, siRNA-mediated silencing of STAT1. **d**, Transwell assay showing the invasion-suppressive effect of SOCS1 in NPC cells, which could be reversed upon simultaneous depletion of STAT1. * *p* < 0.05, ** *p* < 0.01, *** *p* < 0.001 versus NC group. **e**, Wound-healing assay showing the migration-inhibiting effect of SOCS1 in NPC cells, which could be reversed by simultaneous depletion of STAT1. * *p* < 0.05, ** *p* < 0.01, *** *p* < 0.001 versus NC group. **f**, Representative flow cytometry plots showing the pro-apoptotic effect of SOCS1 in NPC cells. * *p* < 0.05, ** *p* < 0.01, *** *p* < 0.001 versus NC group

The SOCS family proteins are the negative-feedback inhibitors of the JAK/STAT signaling pathway [17]. As one of the most potent family members, SOCS1 can inhibit the JAK/STAT pathway either as a ubiquitin ligase by recruiting Cullin5 to signaling components [18] or inhibit directly the kinase activity of JAK [19]. Therefore, we determined the influences of gain- and loss- of SOCS1 (Fig. 4a) on the expression of the JAK/STAT pathway component proteins, respectively. As the western blot results displayed, STAT1 protein was dramatically decreased in the SOCS1-overexpressing NPC cells, while the opposite was true when SOCS1 was silenced (Fig. 4b). Instead, the amounts of other JAK/STAT pathway component proteins including JAK2, JAK3 and STAT3 were barely affected by SOCS1. Mechanistically, SOCS1 specifically interacts with STAT1 but not other proteins in the pathway like JAK2 (Supplementary Figure 1A). The proteasome inhibitor MG132 significantly postponed degradation of both JAK2 and STAT1 in the presence of cycloheximide, indicating they are subject to a control by the ubiquitin-proteasome system. Because JAK2 did not directly interact with SOCS1 (Supplementary Figure 1A), we restricted the mechanistic investigations to STAT1 in the following studies. As expected, STAT1 was undergone a ubiquitination modification in NPC cells as evidenced by the co-immunoprecipitated ubiquitin by STAT1 (Supplementary Figure 1B). Forced expression of SOCS1 not only drastically increased total amount of the ubiquitin-modified STAT1 (Supplementary Figure 1C), but also accelerated STAT1 protein degradation (Supplementary Figure 1D) in both of the tested NPC cell lines.

The JAK/STAT signaling contributes to multiple tumor malignancies by promoting the survival, self-renewal, and metastasis of cancer stem cells [20]. We wondered how does the SOCS1/STAT1 regulatory network affect the NPC cell phenotypes. Obviously, SOCS1-depleted NPC cells exhibited a series of the cancerous propensities including enhanced proliferation (Fig. 4c), invasion (Fig. 4d), migration (Fig. 4e) coupled with decreased spontaneous apoptosis (Fig. 4f) in comparison to the control cells. Of note, simultaneous silencing of STAT1 completely abolished all the stimulative effects in the SOCS1-depleted NPC cells. Taken together, these data indicate SOCS1 plays a role of tumor suppressor in NPC cells by directing the ubiquitin-proteasome-mediated degradation of STAT1 in the JAK/STAT signaling pathway.

### LINC00669 stabilizes STAT1 by holding it back from ubiquitination modification

**Fig. 5:**
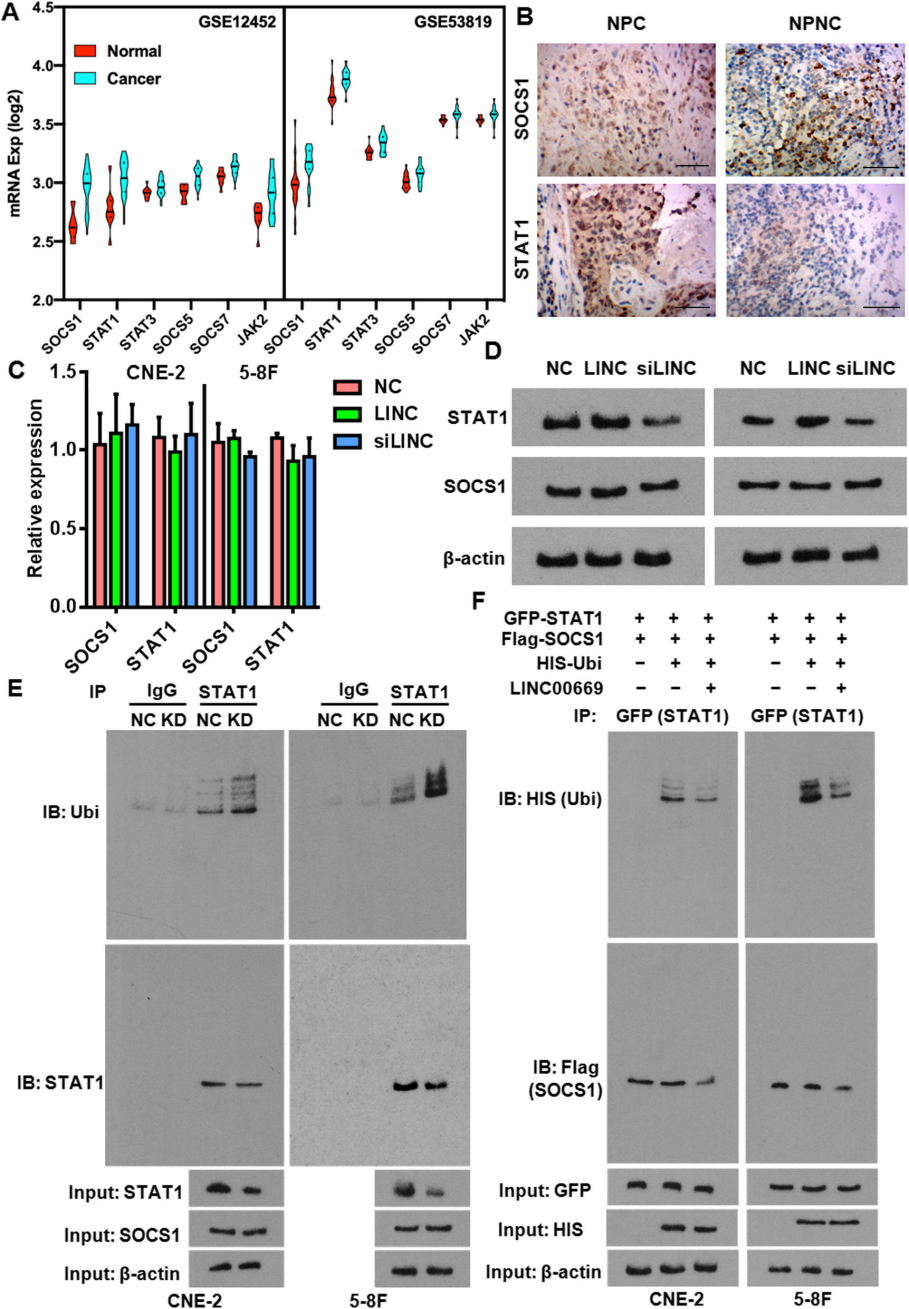
LINC00669 negatively regulates STAT1 ubiquitination. **a**, Microarray data showing upregulation of the key JAK/STAT signaling pathway component genes in NPC tumors. **b**, Representative IHC results showing the aberrantly enriched STAT1 protein in NPC tumors in comparison to nasopharyngeal non cancer (NPNC) tissues. **c**, qPCR analysis showing neither SOCS1 nor STAT1 were subject to a changed with LINC00669 at transcription level. LINC, overexpression of LINC00669; siLINC, siRNA-mediated silencing of LINC00669. **d**, Western blotting showing STAT1 but not SOCS1 protein expression was regulated by LINC00668. Ectopic expression of LINC00669 increased STAT1 protein amount while depletion of LINC00668 decreased STAT1 protein abundance. **e**, Co-IP assay showing endogenous STAT1 ubiquitination was dramatically increased upon knockdown (KD) of LINC00669 in NPC cells. Ubi, ubiquitin. Normal IgG was used as a negative control for IP. **f**, Co-IP assay showing exogenous ubiquitination modification on GFP-fused STAT1 protein was dramatically diminished upon overexpressing LINC00669

Aberrant activation of the JAK/STAT pathway has been found in many tumors [10]. Indeed, most of the component genes of this signaling pathway were upregulated in human NPC tumors at the transcription level (Fig. 5a). Unexpectedly, this was concurrent with upregulation of the negative feedback regulator SOCS1. We further examined the expression of SOCS1 and STAT1 proteins in NPC tumors and para-carcinoma tissues by IHC. Although SOCS1 levels were comparative between the normal and cancerous tissues, it was NPC tumors instead of para-carcinoma tissues that exhibited a strong immunostaining signal of STAT1 (Fig. 5b), indicating the interrupted Inhibition of SOCS1 on STAT1 in NPC tumors. Given the fact that SOCS1 strongly interacts with LINC00669, which is highly expressed in NPC tumors, we investigated how changes of LINC00669 and then its interaction with SOCS1 would affect SOCS1/STAT1 network. Obviously, transcription of SOCS1 and STAT1 did not vary with the expression of LINC00669 (Fig. 5c). Nor did SOCS1 protein change accordingly (Fig. 5d). Instead, STAT1 protein was increased when LINC00669 was overexpressed and was decreased when LINC00669 was depleted (Fig. 5d). Concomitantly, loss of LINC00669 also led to an increase in ubiquitination of STAT1 (Fig. 5e), which could be reversed upon forced expression of LINC00669 (Fig. 5f).

**Fig. 6:**
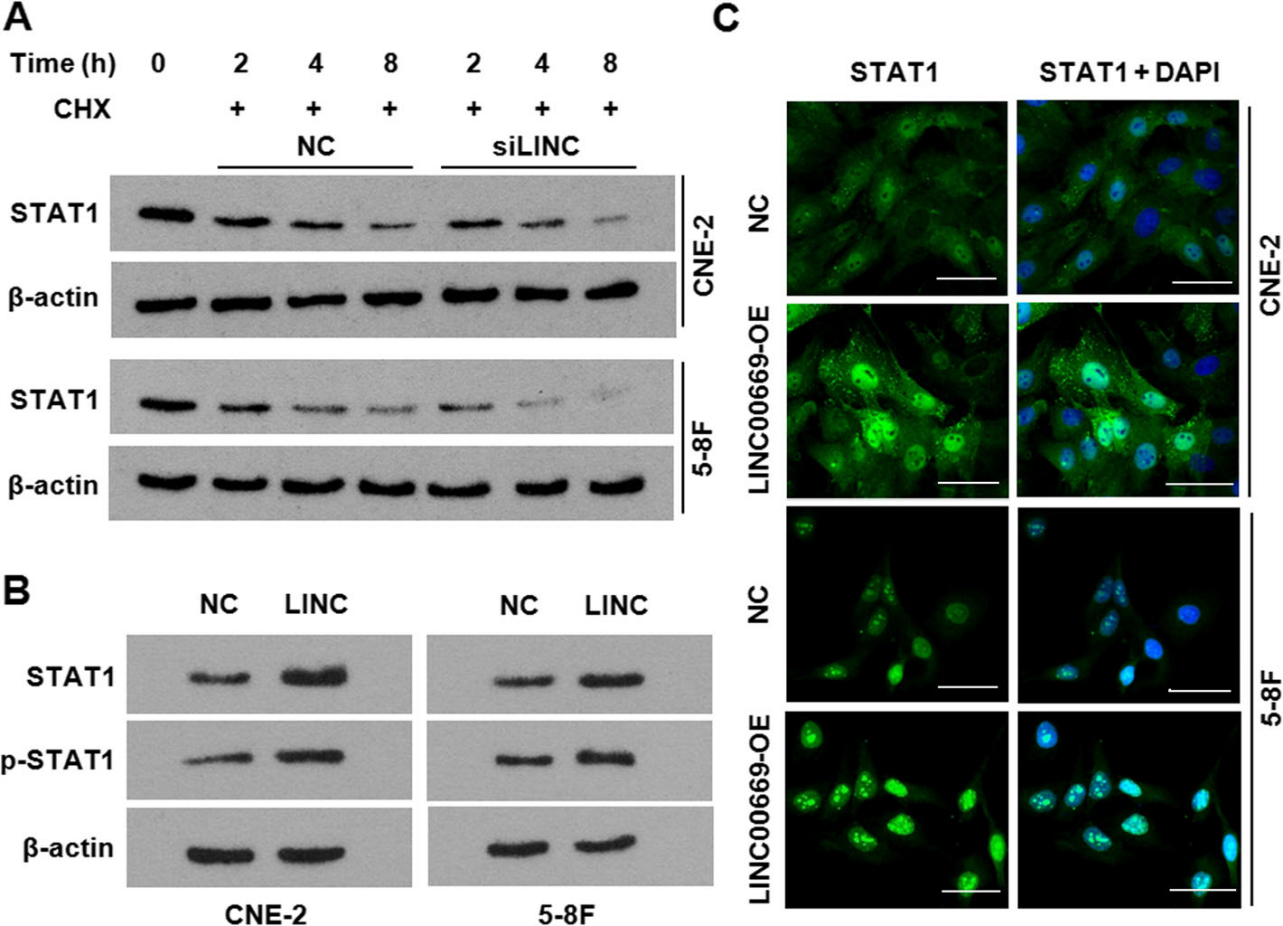
LINC00669 stabilizes STAT1 and facilitates its nuclear translocation. **a**, Western blotting showing the decreased STAT1 protein stability in the absence of LINC00669 in NPC cells. NC, negative control; siLINC, siRNA-mediated silencing of LINC00669; CHX, cycloheximide. **b**, Western blotting showing phosphorylation (Y701) of STAT1 was greatly enhanced upon ectopic expression of LINC00669. **c**, Immunofluorescence assay showing the increased nuclear translocation of STAT1 in the LINC00669-everexpressing NPC cells

As SOCS1 destabilizes STAT1 through the ubiquitin-proteasomal protein degradation, we postulated that changes in LINC00669 expression might also lead to the altered STAT1 protein stability in view of the apparent change in the ubiquitination modification it brought to STAT1 (Fig. 5e, f). Indeed, depletion of LINC00669 largely shortened the half-life of STAT1 as evidence by the accelerated degradation of STAT1 protein in both of the cycloheximide-treated CNE-2 and 5-8F cells (Fig. 6a). Interestingly, not only the total amount of STAT1, but also the phosphorylated STAT1 was greatly increased as a consequence of overexpressing LINC00669 (Fig. 6b), which expectedly led to a nuclear translocation of STAT1 in NPC cells (Fig. 6c).

### Removal of SOCS1 reverses the defective phenotypes of LINC00669-depleted NPC cells in vitro and in vivo

**Fig. 7:**
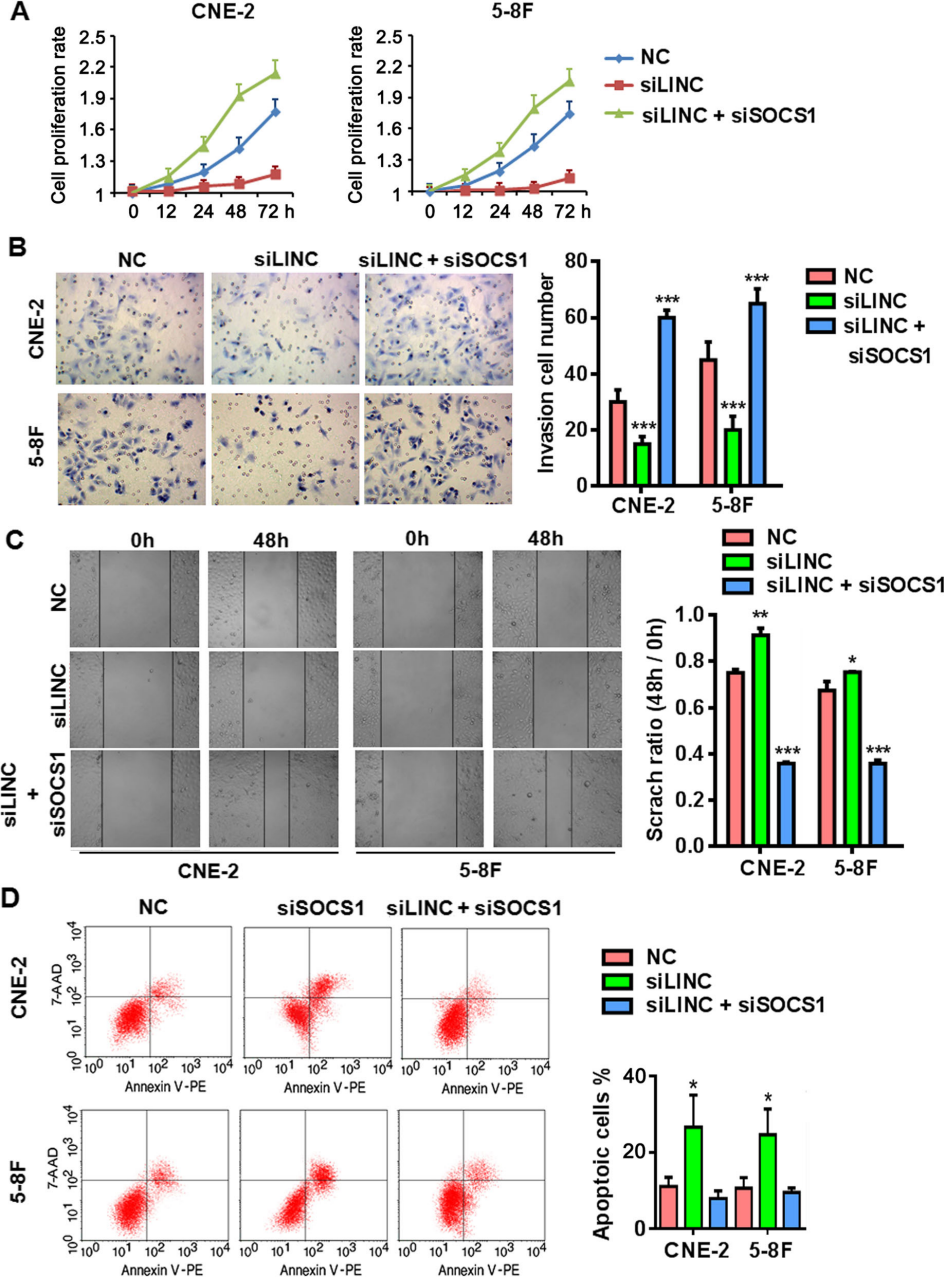
SOCS1 is indispensable for oncogenic function of LINC00669 in NPC cells. **a**, MTT assay showing knockdown of SOCS1 partially rescued the time-dependent proliferation defect in the LINC00669-depleted NPC cells. NC, negative control; siLINC, siRNA-mediated silencing of LINC00669; siSOCS1, siRNA-mediated silencing of SOCS1. **b**, Transwell assay showing reverse of the impaired invasion by knockdown of SOCS1 in the LINC00669-depleted NPC cells. * *p* < 0.05, *** *p* < 0.001 versus NC group. **c**, Wound-healing assay showing restoration of the migrative defect by knockdown of SOCS1 in the LINC00669-depleted NPC cells. * *p* < 0.05, ** *p* < 0.01, *** *p* < 0.001 versus NC group. **d**, Representative flow cytometry plots showing knockdown of SOCS1 abolished the massive apoptosis in the LINC00669-depleted NPC cells. * *p* < 0.05, ** *p* < 0.01, *** *p* < 0.001 versus NC group

Now that the establishment of LINC00669/SOCS1/STAT1regulatory cascade, we wondered whether this could be the mechanism that underlies the profound influences of LINC00669 and SOCS1 on the featured NPC cell phenotypes. As expected, silencing LINC00669 suppressed NPC cell proliferation (Fig. 7a), invasion (Fig. 7b) and migration (Fig. 7c), and induced spontaneous apoptosis (Fig. 7d). Simultaneous depletion of SOCS1 completely abolished all the detrimental effects observed in the LINC00669-silenced NPC cells.

**Fig. 8:**
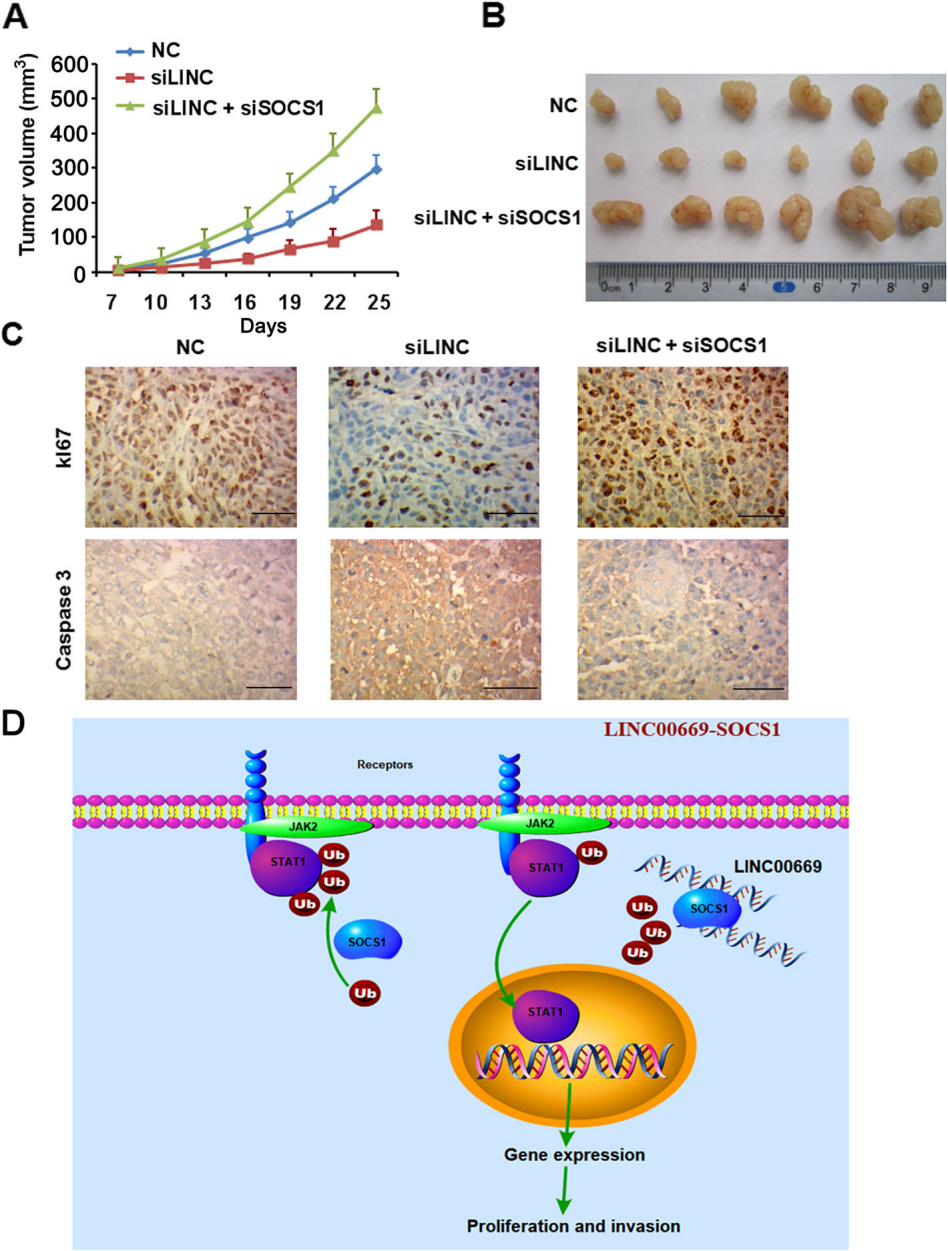
SOCS1 is indispensable for tumor-promoting function of LINC00669 in vivo*.* **a**, Tumor growth curves as recorded by measuring tumor volume. Depletion of LINC00669 hampered xenograft tumor growth in nude mice, which could be partially rescued by simultaneous knockdown of SOCS1 in NPC cells. NC, negative control; siLINC, siRNA-mediated silencing of LINC00669; siSOCS1, siRNA-mediated silencing of SOCS1. **b**, Representative images showing xenograft tumors. **c**, IHC results showing the number of proliferating (Ki67^+^) cells was decreased in tumors derived from LINC00669-depleted NPC cells, where the number of apoptotic (Caspase 3^+^) cells was increased accordingly. In contrast, simultaneous knockdown of SOCS1 in the LINC00669-depleted NPC cells restored cell proliferation and suppressed apoptosis in the xenograft tumors. **d**, The activation of JAK/STAT signaling pathway is under tight control of SOCS1 in normal cells. The growth homeostasis is interrupted due to the aberrant expression of LINC00669. Mechanistically, abnormal accumulation of cytosolic LINC00669 competitively binds to SOCS1, and insulates it from touching the transcription factor STAT1 for ubiquitination modification, which stabilizes STAT1 and promotes further phosphorylation by escaping from the proteasomal degradation. The activated STAT1 then enters the nuclei and initiates the transcriptional program associated with cell proliferation and invasion

Finally, we accessed the role of LINC00669/SOCS1/STAT1 regulatory network in NPC carcinogenesis in vivo. CNE-2 cells with stable knockdown of LINC00669 or concomitant depletion of LINC00669 and SOCS1 were subcutaneously injected into nude mice, respectively. Comparing to the mice injected with control CNE-2 cells, silencing LINC00669 dramatically suppressed xenografted tumor growth (Fig. 8a, b). Instead, tumor growth retardation shown in mice bearing tumors derived from LINC00669-deficient CNE-2 cells was completely reversed in the recipients injected with LINC00669 and SOCS1 double knockdown cells. In accordance, the proliferative cells in xenograft tumors derived from LINC00669 silencing CNE-2 cells were largely diminished compared to the control tumors, while tumors grown from LINC00669 and SOCS1 double knockdown CNE-2 cells were overwhelmed with massive of Ki67 positive cells (Fig. 8c). In contrast, apoptosis as indicated by Caspase 3 immunostaining appeared in an opposite pattern to that featured in proliferation (Fig. 8c).

As a whole, our study revealed a novel tumor-promoting mechanism involving LINC00669/SOCS1/STAT1 regulatory network in NPC (Fig. 8d). Briefly, LINC00669 is aberrantly increased in the cytoplasm of NPC cells, where it binds to and prevents SOCS1 from imposing ubiquitination modification on the JAK/STAT signaling pathway component transcription factor STAT1. As a result, total and phosphorylated STAT1 are accumulated by escaping from proteasomal degradation. Phosphorylation of STAT1 facilitates its nuclear translocation and initiation of the transcriptional program for the enhanced proliferation and invasion.

## Discussion

Normal cell functions are directed by the finely tuned activation and inactivation of diverse cellular signaling pathways. The JAK-STAT signaling pathway is a chain of interactions between proteins in a cell, and is involved in fundamental processes such as immunity, cell division and cell death [21]. One of the features of this pathway is characterized by the rapid activation and inactivation [22]. Activated STATs accumulate rapidly in the nucleus, and within a few hours, the inactivated STATs return to the cytoplasm and prepare for the next round of signaling. Disruption of the cellular machineries that control the JAK-STAT signaling may lead to a variety of diseases, such as skin conditions, cancers, and disorders in the immune system [23]. In this study, we revealed that LINC00669 participates in regulating the JAK-STAT signaling pathway negative feedback loop of SOCS1/STAT1, through which it plays an oncogenic role in NPC tumorigenicity.

The localization of lncRNAs within a cell is the primary determinant of their molecular functions. Cabili and colleagues used FISH to directly visualize the regional RNA expression and observed the complex localization patterns of 34 lncRNAs, which can be divided into five categories: large nuclear foci, large nuclear foci with single molecules scattered through the nucleus, predominantly nuclear without foci, cytoplasmic and nuclear, and predominantly cytoplasmic [24]. Generally, nuclear lncRNAs act as chromatin-restricted regulators of gene transcription and chromatin structure [25–28]. Instead, sufficient evidence shows that cytoplasmic localized lncRNAs perform parts suitable for the cytoplasm including translational regulation, signaling, and respiration. For example, lncRNAs interacting with microRNA are enriched in the cytoplasm [29, 30]. In addition, lncRNAs that control protein stability or mRNA translation efficiency are also more common in the cytoplasm and ribosomes [31–33]. In agreement to these assumptions, LINC0069, which is cytoplasmically enriched in NPC cells, does not directly set up cancerous transcriptome, but to regulate the JAK/STAT signaling pathway through the physical binding to inactivate its key negative feedback regulator SOCS1 in the cytoplasm.

SOCS proteins are potent JAK/STAT signaling pathway suppressors. All SOCS family members are featured with a SH2 domain and a brief C-terminal domain, namely SOCS box1 that is physically associated with the adaptor complex, elonginBC2 [34, 35]. This connection facilitates the ubiquitination of signal intermediates by recruiting the E3 ubiquitin ligase scaffold (Cullin5) [18]. Except for the common ubiquitin ligase activity, SOCS1 and SOCS3 are capable of a direct inhibition on JAK kinase activity through a short motif called Kinase Inhibitory Region [18], which is however, unlikely true in NPC cells in that lacking of the direct interaction between SOCS1 and JAK. Instead, SOCS1 directs proteasomal degradation of STAT1 by catalyzing ubiquitination on the protein.

Constitutive activation of STAT1 and STAT3 has been reported in NPC tissues [36]. However, the abnormal STAT3 activity in NPC should not be ascribed to the aberrant expression of LINC00669 since it remained stable regardless of the changes in LINC00669 expression. Instead, STAT1 is highly responsive to the cytosolic content of LINC00669, whose ectopic expression leads to increases in both total and phosphorylated STAT1. Inhibition of STAT1 can enhance the radiosensitivity of CNE-2R cells, and knockout STAT1 causes cell growth retardation and apoptosis both in vitro and in vivo [37]. Therefore, we consider STAT1 as a key effector in LINC00669/SOCS1/JAK-STAT signaling cascade, which compiles cancerous transcriptome in NPC cells.

In conclusion, although it has been aware of the aberrant activation of the JAK/STAT signaling pathway in NPC tumors for long, its causes remain largely underexplored. We revealed in current study that lncRNA LINC00669 was upregulated in NPC cell cytoplasm, where it binds to SOCS1, and blocks its ubiquitination modification function toward STAT1. Interruption of such negative feedback machinery enhances STAT1 stability and facilitates its phosphorylation and thus nuclear translocation to initiate the proliferation and invasion-associated transcriptome. Our study not only provides a novel marker for diagnosis and prognosis of NPC, but also highlights it a potential target for therapy.

## Supplementary information

**Additional file 1.**

## Data Availability

The datasets generated/analyzed in the present study are available upon reasonable request from the corresponding author.

## Abbreviations

Co-IP: Co-immunoprecipitation
DAB: Diaminobenzidene
EBV: Epstein-Barr virus
FBS: Fetal bovine serum
FISH: Fluorescence in situ hybridization
HOTAIR: HOX transcript antisense intergenic RNA
IHC: Immunohistochemistry
JAK: Janus kinase
lncRNA: long non-coding RNA
MEG3: Maternally expressed gene 3
NPC: Nasopharyngeal carcinoma
Nt: Nucleotides
qPCR: quantitative PCR
RIP: RNA immunoprecipitation
STAT: Signal transducer of activators of transcription
