# Supplementary figures and images for: LINC00669 insulates the JAK/STAT suppressor SOCS1 to promote nasopharyngeal cancer cell proliferation and invasion
Source: J Exp Clin Cancer Res. 2020 Aug 24;39:166. doi: 10.1186/s13046-020-01674-z (PMC7444085; doi:10.1186/s13046-020-01674-z)

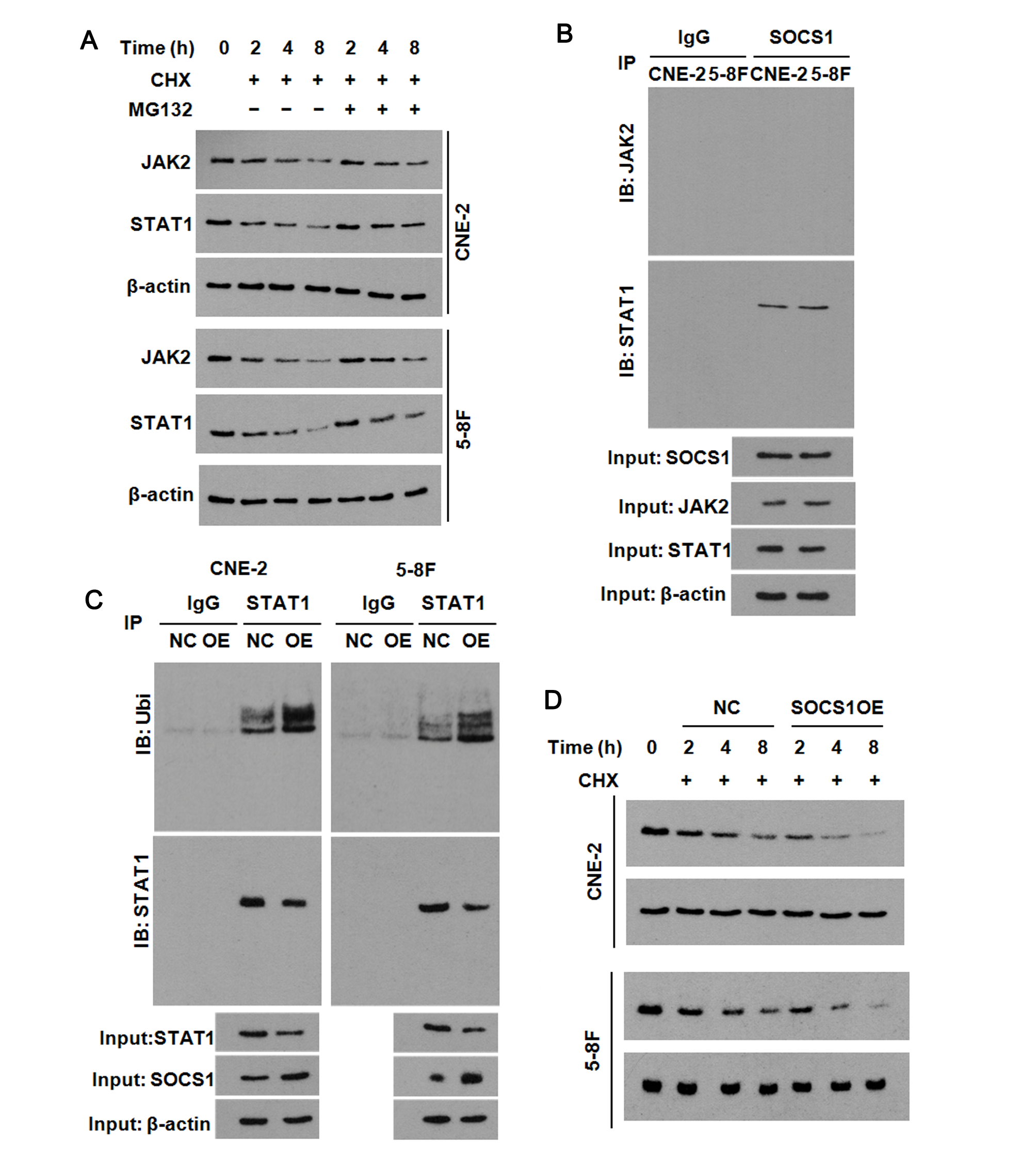

Supplement: Supplementary file 1 — Additional file 1. [file 13046_2020_1674_MOESM1_ESM.tif]
